# Supplementary figures and images for: Gene-based association study for lipid traits in diverse cohorts implicates BACE1 and SIDT2 regulation in triglyceride levels
Source: PeerJ. 2018 Jan 29;6:e4314. doi: 10.7717/peerj.4314 (PMC5793713; doi:10.7717/peerj.4314)

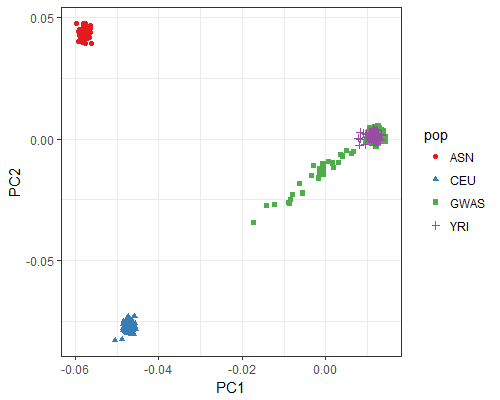

Supplement: Figure S1 — The first two genotypic principal components for the Yoruba lipid cohort (GWAS) are plotted in comparison to HapMap populations YRI (Yoruba in Ibadan, Nigeria), CEU (European ancestry in Utah), and ASN (Chinese in Beijing and Japanese in Tokyo). [file peerj-06-4314-s005.png]

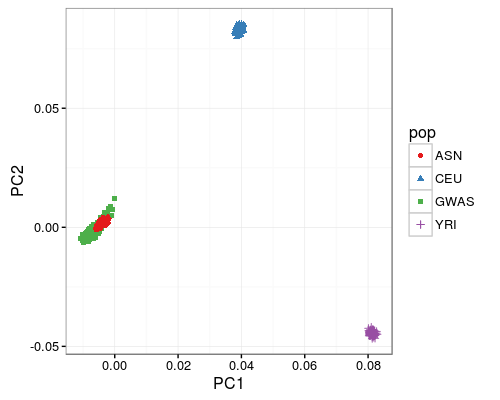

Supplement: Figure S2 — The first two genotypic principal components for the Cebu lipid cohort (GWAS) are plotted in comparison to HapMap populations YRI (Yoruba in Ibadan, Nigeria), CEU (European ancestry in Utah), and ASN (Chinese in Beijing and Japanese in Tokyo). [file peerj-06-4314-s006.png]
